# Attitudes of women towards products containing hormones (hormonal contraceptives or hormone therapy): what changes from pre to postmenopause?

**DOI:** 10.1080/07853890.2021.1938662

**Published:** 2021-06-14

**Authors:** Giovanni Grandi, Maria Chiara Del Savio, Valentina Boggio Sola, Francesca Monari, Chiara Melotti, Fabio Facchinetti

**Affiliations:** Department of Medical and Surgical Sciences for Mother, Child and Adult, Azienda Ospedaliero-Universitaria di Modena, University of Modena and Reggio Emilia, Modena, Italy

**Keywords:** Hormonal contraceptives, postmenopausal hormone therapy, thrombosis, venous thromboembolism, depression, breast cancer, cervical cancer, ovarian cancer

## Abstract

**Introduction:**

To evaluate the actual perceptions of hormonal contraceptives (HC) in women of reproductive age in comparison with similar concerns of postmenopausal women in relation to hormone therapy (HT).

**Materials and methods:**

A questionnaire-based study was conducted in a population of 370 women, 198 (53.5%) premenopausal and 172 (46.5%) postmenopausal. Perceptions were evaluated using specific questions and Likert scales (-5 to +5).Multivariate regression analyses were adjusted for categories of HC/HT use (never, past and current).

**Results:**

The most reported adverse effect associated with hormonal treatments was venous thrombosis (1.4 ± 0.1), especially for HC use in premenopausal women (*p* < .0001). According to responses, hormonal treatments can increase the risk of developing venous thrombosis (OR 0.79; 95% CI 0.67–0.96, *p* = .004) and depression (OR 0.80; 95% CI 0.69–0.92, *p* = .002) more in pre-menopause, while they can increase the risk of weight gain more in post-menopause (OR 1.24: 95% CI 1.07–1.42, *p* = .003).The greatest oncological concern throughout life was about breast cancer (0.9 ± 0.1). There was the perception that hormonal treatments can increase the risk of developing ovarian cancer more in post-menopause (OR 1.44; 95% CI 1.19–1.75, *p* = .0002), while they can increase the risk of uterine cervix cancer more in pre-menopause (OR 0.74; 95% CI 0.52–0.97, *p* = .03).

**Conclusions:**

Our data suggest that our patients are primarily concerned about the effects of hormonal treatments on venous thrombosis, mood, breast cancer and cervical cancer risk, and, later in life, about their impact on weight gain, breast and ovarian cancer risk.

**KEY MESSAGES**
1. Young patients are primarily concerned about the effects of hormonal treatments on venous thrombosis, mood, breast cancer and cervical cancer risk.
2. Older patients are primarily concerned about the effects of hormonal treatments on weight gain, breast and ovarian cancer risk.
3. The greatest oncological concern throughout life was about breast cancer.

## Introduction

Nowadays, hormonal treatments are commonly used by millions of women worldwide. Most women use at least one hormone therapy at some point of their life in the developed world, including hormonal contraceptives (HC) and hormone therapy (HT) after menopause. Despite being widely-prescribed, users are still doubtful about synthetic hormones, both in pre- and post-menopause.

More than 9 million premenopausal women using contraception relied on the contraceptive pill in 2014 in the US [1,2]. In Europe, considering countries such as France, Spain, Germany, Italy and United Kingdom, around 22 million out of 72 million premenopausal women are currently using of contraceptive pill [3], with rates of usage varying from 35% in Spain to 63% in Germany [4]. Available contraceptives make it possible to choose between combined formulations based on oestrogens and progestin and those containing only a progestin. The former varies in doses and types of oestrogens or progestins, as well as route of administration (pill, patch, vaginal ring, subcutaneous implant, injection or intrauterine device). HC use in premenopausal women plays not only the role of preventing unintended pregnancies [5]: the physiological actions of the oestrogens and progestins also provide important non-contraceptive benefits, including treatment of common gynaecological and non-gynaecological medical conditions [6].

Indications for hormone therapy are not limited to women of reproductive age: hormones are also widely used in post-menopausal women, with different therapeutic schemes. For example, HT is used to relieve menopausal symptoms [7]. It is the principal therapy for urogenital and vasomotor symptoms of menopause, offering multiple additional beneficial effects on women’s health, such as prevention and management of osteoporosis [8] and dementia [9,10]. Furthermore, as confirmed by the Women’s Health Initiative (WHI) study, it can exert a protective effect on the cardiovascular system if started at the beginning of menopause [7,8] and can also improve sleep and mood disorders [8–10]. There are different formulations for postmenopausal HT: oestrogen only (indicated for women who have previously undergone a hysterectomy), oestrogen combined with progestin (indicated for women who have not previously undergone a hysterectomy), and “progestin-free” drugs, such oral tibolone and combinations of oestrogen with bazedoxifene (Tissue Selective Oestrogen Complex) [11]. HT can be taken at different doses and in different ways: orally, as a patch, or as a transdermal [8].

The indications for hormone therapies, both pre- and post-menopausal, are many, as are the concerns about them. Deepening the conception that women of all ages have about hormone therapies can help in the counselling that should guide the personalized choice of drugs. For these reasons, in this study we want to investigate the current knowledge of the benefits and risks of hormonal treatments by comparing responses to an identical set of questions from women of reproductive age and postmenopausal women.

## Methods

### Study design

This is a cross-sectional, observational study performed from December 2019 to May 2020 at the Department of Obstetrics and Gynaecology at Azienda Ospedaliero-Universitaria of Modena (Italy) in premenopausal or postmenopausal subjects evaluated at a routine visit.Included subjects were a group of women with no previous oncological diseases evaluated for a routine visit at the general gynecological service of the hospital (“Ambulatorio Divisionale”). This service is where general practitioners send patients for routine gynecological examinations. Therefore, they are in general healthy women who did not present particular gynecological disorders or complaints. Menopausal status was classified as amenorrhoea for over 12 months.

### Evaluated variables

After detailed counselling about the study, the women who chose to participate signed an informed consent form. Once included in the study, women were given an identical questionnaire depending on the group they belonged to (pre- or post-menopause) (i.e. use of HC or HT) (Supplemental Table 1). The questionnaires were self-administered before the medical consultation, without intervention of the researchers collecting the data. During the gynaecological examination, clinical data from the included women were collected: age, parity, and number of vaginal or caesarean births, abortions, and gynaecological surgeries previously performed.

**Table 1.** Features of *n* = 370 women included in the study.

	Premenopausal (*n* = 198)	Postmenopausal (*n* = 172)	*p*
Age (years old)	32.3 ± 8.9	54.7 ± 7.1	<.0001
Nulliparous (*n*, %)	144 (72.7%)	26 (15.1%)	<.0001
HC use (*n*, %)			
Present users	65/198 (32.8%)	/	
Past users	95/198 (48.0%)	117/172 (68.0%)	
Never	38/198 (19.2%)	55/172 (32.0%)	
Actual duration of HC use (years)	3.6 ± 3.5	/	
Past duration of HC use (years)	4.7 ± 4.4	7.5 ± 7.7	
HT use (*n*, %)			
Present users	/	19/172 (11.0%)	
Past users	/	23/172 (13.4%)	
Never	/	130/172 (75.6%)	
Actual duration of HT use (years)	/	1.7 ± 1.8	
Past duration of HT use (years)	/	3.9 ± 4.7	

HC: hormonal contraception; HT: postmenopausal hormone therapy.

For both groups of women, questionnaires collected data on the type of HC/HT eventually used, the brand name of the product, use (current, past, never used) and the duration of use (months/years). Women were asked to evaluate how much in their opinion HC/HT could affect the risk of developing some forms of cancer (breast, ovary, colon, uterine body, uterine cervix) or diseases (venous thrombosis, breast cysts, cardiovascular diseases, depression), or other classical possible side-effects (headache, weight gain, reduced sexual desire, vaginal dryness, increased appetite, mood swings) using a Likert scale from −5 to +5. For cancers and diseases, −5 = reduces the risk of onset, 0 = neutral effect, +5 = increases the risk of onset, while for symptoms, −5 = worsens with HT, 0 = neutral effect, +5 = improves with HT (Supplemental Table 1). For the final analyses, included women were divided according to their HC and/or HT use in 3 categories: 1) never users (women who had never used HC and HT) 2) past users (women using HC and/or HT in the past) or 3) current users (women who are taking HC or HT at the moment of the study inclusion).

An English translation of the specific questionnaires used in this study are reported in Supplemental Table 1. The types of questions were obtained from a previously published questionnaire-based study undertaken in the same area of Italy [12] and another study recently published by our research group [13].

### Ethics and statistics

The study was designed and conducted in full accordance with the World Medical Association Declaration of Helsinki, as revised in 2002. The ethics committee of Modena approved the use of the questionnaires in our Department (reference 36/17) and a specific informed consent was obtained from each woman for the use of her sensitive data in research analysis.

The questionnaire responses of pre- and post-menopausal women were analyzed and compared. The frequency of answers in the different groups was calculated. When necessary, the prevalence was compared using contingency tables. Comparisons of continuous variables between groups were performed using Student's *t* test. Multivariate logistic and multiple regression analysis were used to investigate risk factors associated with different perceptions in pre or postmenopause, adjusting for confounding such as categories of HC/HT use (never, past and current): candidate variables were included if significant on univariate analysis or clinically relevant. Multiple regression models were used to study the impact of women age. Statistical analysis was performed using the statistical package StatView (v 5.01.98; SAS Institute Inc, Cary, NC, USA). Correlations were considered significant at a *p*-value <.05. Continuous results are expressed as mean ± standard deviation (SD).

### Sample size calculation

Assuming a pooled SD of 1 unit, the study was determined to require a sample size of 128 women for each group (giving a total sample size of 256, assuming equal group sizes) to achieve a power of 80% and a level of significance of 5% (two sided), for detecting a true difference in means of 0.35 units in Likert Scale values between different groups.

## Results

### Study group

A population of 370 women (mean age: 42.7 ± 13.8 years, range 19.0–74.0 years) was included in the study and completed the specific questionnaires. Of these women, 198 (53.5%) were premenopausal, while 172 (46.5%) were postmenopausal. The features of the two groups and their current/past use of HC and/or HT are reported in Table 1. According to categories about HC/HC use (see above), included women can be divided in *n* = 84 never users, *n* = 202 past users and *n* = 84 current users.

### Side effects of hormonal treatments

#### Knowledge of effects of hormonal treatment on disease development and side effects

The answers to the questions “How much can HC/HT increase or reduce the risk of developing these diseases?” and “How much can HC/HT improve or worsen these symptoms?” are reported in Table 2, for the whole study group and for pre- and post-menopausal women separately.

**Table 2.** Mean ± Standard Error (Likert scale from -5 to +5) sort in descending order to the questions “How much does HC/HT increase or reduce the risk of developing these diseases?” and “How much does HC/HT improve or worsen these symptoms?” for the whole study group and for premenopausal vs. postmenopausal women, separately.

How much does HC/HT increase or reduce the risk of developing these diseases?				
	Total group (*n* = 370)	Premenopausal women (*n* = 198)	Postmenopausal women (*n* = 172)	*p*
Venous thrombosis	1.4 ± 0.1	1.8 ± 0.2	0.8 ± 0.2	**<.0001**
Breast cysts	0.6 ± 0.1	0.5 ± 0.1	0.7 ± 0.2	.33
Cardiovascular diseases	0.5 ± 0.1	0.9 ± 0.1	0.1 ± 0.2	**.0003**
Depression	0.0 ± 0.1	0.6 ± 0.1	−0.6 ± 0.2	**<.0001**
How much does HC/HT improve or worsen these symptoms?				
Headache	0.4 ± 0.1	0.3 ± 0.2	0.5 ± 0.2	.37
Mood swings	0.4 ± 0.1	0.5 ± 0.2	0.1 ± 0.2	.15
Weight increase	0.3 ± 0.1	0.0 ± 0.2	0.7 ± 0.2	**.02**
Increased appetite	0.3 ± 0.1	0.4 ± 0.1	0.3 ± 0.2	.82
Vaginal dryness	0.0 ± 0.1	−0.1 ± 0.1	0.1 ± 0.2	.36
Reduction of libido	−0.1 ± 0.1	−0.1 ± 0.1	−0.1 ± 0.2	.78

Bold characters represent statistically significant results (*p* < .05).

The most reported adverse effect associated with hormonal treatments was venous thrombosis (1.4 ± 0.1), especially for HC use in premenopausal women (*p*<.0001). Similarly, a negative effect on cardiovascular diseases is reported more frequently in premenopausal women (*p*=.0003) (Table 2). On the other hand, a possible negative impact on depression was reported only for HC use in premenopausal women (*p*<.0001) (Table 2).

Interestingly, the most significant side effects of HC/HT use were headache (0.4 ± 0.1) and mood swings (0.4 ± 0.1). The impact of HT on weight increase was significantly overestimated by postmenopausal women (*p* = .02) (Table 2).

Categories of HC/HT use (never, past, current users) were not linked to knowledge of effects of hormonal treatment on disease development and side effects (*p*>.05), both in pre and in postmenopause.

#### Multivariate analysis

##### Menopausal status

Results of the multivariate analysis are reported in Table 3 and confirm the presented results. According to the responses, hormonal treatments can increase the risk of developing venous thrombosis and depression more in premenopausal women, while they increase the risk of weight gain more in post-menopause.

**Table 3.** Multivariate logistic regression analyses.

	Perceived OR of HC/HT can increase the risk of developing these diseases (postmenopausal vs. premenopausal women)	*p*
Venous thrombosis	0.79 [0.67–0.93]	.004
Depression	0.80 [0.69–0.92]	.002
	Perceived OR of HC/HT can worsen this symptom (postmenopausal vs. premenopausal women)	
Weight increase	1.24 [1.07–1.42]	.003
	Perceived OR of HC/HT can increase the risk of developing these cancers (postmenopausal vs. premenopausal women)	
Ovarian cancer	1.44 [1.19–1.75]	.0002
Uterine cervix cancer	0.74 [0.52–0.97]	.03

##### Age

Calculations based on the age of included women confirmed the results of menopausal status. In a multiple regression model including all of the evaluated diseases, age was significantly inversely associated with the response to the question “HC/HT can increase the risk of developing venous thrombosis (coefficient −1.13, *p*=.005) and depression” (coefficient −1.16, *p* = .001) (i.e. greater belief that there is an increase in risk in younger women).

In a multiple regression model including all possible symptoms associated with treatment, age was significantly associated with the response to the question “HC/HT can increase the risk of weight increase” (coefficient +0.87, *p*=.01) (i.e. greater belief that there is an increase in risk in older women).

### Oncological effects of hormonal treatments

#### Knowledge of effects of hormonal treatments on cancer development

The answers to the question “How much can HT increase or reduce the risk of developing these cancers?” are reported in Table 4. In general, the greatest concern was about breast cancer (0.9 ± 0.1), followed by uterine cervix cancer (0.4 ± 0.1) and uterine body cancer (0.2 ± 0.1) (Table 4). The protective effect on ovarian cancer was only significantly reported concerning HC in premenopausal women (*p*<.0001). Also, the perceived protective role of HC or HT on colorectal cancer was more evident for HC in premenopausal women (*p*=.03).

**Table 4.** Mean ± Standard Error (Likert scale from -5 to +5) sort in descending order to the question “How much does HC/HT increase or reduce the risk of developing these cancers?” for the whole study group and for the whole study group and for premenopausal vs. postmenopausal women, separately.

How much does HC/HT increase or reduce the risk of developing these cancers?				
	Total group (*n* = 370)	Premenopausal women (*n* = 198)	Postmenopausal Women (*n* = 172)	*p*
Breast cancer	0.9 ± 0.1	0.7 ± 0.2	1.1 ± 0.2	.21
Uterine cervix cancer	0.4 ± 0.1	0.2 ± 0.1	0.5 ± 0.2	.33
Uterine body cancer	0.2 ± 0.1	0.0 ± 0.2	0.5 ± 0.2	**.04**
Ovarian cancer	0.0 ± 0.1	−0.6 ± 0.1	0.6 ± 0.2	**<.0001**
Colorectal cancer	−0.2 ± 0.1	−0.4 ± 0.1	−0.1 ± 0.2	**.03**

Bold characters represent statistically significant results (*p* < .05).

Categories of HC/HT use (never, past, current users) were not linked to knowledge of effects of hormonal treatments on cancer development (*p*>.05), with the only exception of the effect on ovarian cancer: current users were significantly more reporting a protective effect (*p*=.001).

#### Multivariate analysis

##### Menopausal status

Results of the multivariate analysis are reported in Table 3. There is the perception that hormonal treatments increase the risk of developing ovarian cancer more in postmenopausal women, whereas they increase the risk of uterine cervix cancer more in premenopausal women.

##### Age of women

In a multiple regression model including all evaluated cancers, age was significantly associated with the response to the question “HC/HT can increase the risk of developing ovarian cancer” (coefficient 2.18, *p*<.0001) (i.e. greater belief that there is an increase in risk in older women), and inversely associated with the response to the question regarding “developing uterine cervix cancer” (coefficient −1.95, *p*=.002) (i.e. greater belief that there is an increase in risk in younger women).

## Discussion

### Overall results

The results of the present study should be used to guide counselling when prescribing hormonal treatments, such as HCs in premenopausal women or HTs in postmenopausal women. Addressing patients’ belief in false myths and trying to overcome their more or less justified worries is one of the most important tasks in the therapeutic prescription process today, especially for hormonal treatments.

Our original results demonstrate that premenopausal women tend to overestimate the negative impact of HC on venous thromboembolism and on depression in comparison to the views of post-menopausal women surrounding HT. Conversely, postmenopausal women are much more concerned about the possible weight gain associated with HT.

Regarding the oncological risk of these therapies, we have demonstrated that fear of breast cancer is similar in pre and postmenopausal women, presenting the most intense life-long oncological worry.

The protective effect on ovarian cancer was correctly reported only by premenopausal women, surrounding HC use, while the protective role of hormonal treatment on colorectal cancer was more evident for HC in premenopausal women (but was also true for HT in postmenopausal women). In particular, we have shown a direct increase in fear surrounding the use of hormones with age, for ovarian cancer (more perceived risk in older women), and an inverse relationship for uterine cervix cancer (more perceived risk in younger women).

### Interpretation

Adequate counselling surrounding hormonal treatments during life may help women recognize the health benefits and risks of these drugs [12]. In daily practice, myths and taboos regarding side effects and long-term consequences of hormonal treatment use on women’s health need to be fully addressed. This partial and summary information transcends continents and stage of life (pre- vs. post-menopause), and is shared by both highly educated women and women with little education [12]. Attitudes of health-care professionals (HCPs) may contribute significantly to women’s knowledge, depending on their ability to share conclusive information with potential users of hormonal treatments.

Most vague complaints attributed to hormonal treatments can be explained by women's negative expectations (the nocebo phenomenon) or by the high background prevalence of such complaints [14]. Moreover, it has been demonstrated that the elderly may be more susceptible to the nocebo phenomenon than the young [15].

Conventional wisdom has been to counsel women thoroughly about hormonal treatments. This practice assumes that anticipatory counselling about side effects will improve acceptability and compliance. However, nocebo phenomenon studies in other areas of medicine suggest that such counselling may have a paradoxical effect: by raising these issues, clinicians may create or aggravate side effects that would not otherwise have occurred [15].

Data on women’s knowledge of the effects of hormonal treatments (both HC and HT) on health and wellbeing are generally very scarce in the literature. Our results concerning venous thrombosis risk are surprising: the perceived risk is more evident in pre- than in post-menopause. Although venous thrombosis incidence naturally increases during the reproductive period of a woman’s life, the difference associated with HC use during this period is relatively low when looking at the whole lifespan; however, the risk during menopause is considerably higher, especially during HT treatment [16,17].

It is well known that hormones and their fluctuations in the natural menstrual cycle are frequently associated with mood alterations: in the context of negative reports regarding HC and mood, the findings of high-quality prospective studies show that the effect is negligible and the effect of hormones on mood is individual [18]. However, as we have demonstrated, the problem of depression associated with hormonal treatments is more felt in young women than older ones.

Communicating cancer risk information is relevant to a number of HCPs. Moreover, information about risk can also be important in motivating individuals to engage in cancer screening. Reliable epidemiological assessment of any association of hormone therapy use with cancer requires large numbers and careful control of all potential sources of appreciable bias.

HC use has demonstrated a clear and significant protective effect on the risk of ovarian cancer. The risk of endometrial cancer is reduced by about 50% in ever users of HCs, a benefit which is greater with increasing duration of use. An association has been found between increased risk of uterine cervical cancer and long-term HC use. How breast cancer risk changes during HC use remains unclear: if an increased risk exists, it is mainly confined to current and very young users of HC [19]. Conversely, from the introduction of post-menopausal HT, there has been great concern that it may increase the risk of breast cancer, particularly in the case of combined HT including a progestin [20,21].Colorectal cancer is the most frequent neoplasm in non-smokers of both sexes combined in Western countries, and HT use seems to reduce its risk [22]. On the other hand, ovarian cancer risk is significantly increased in current users of HT, even in those with less than 5 years of HT use [23].

Breast cancer is the most common cancer diagnosed among women in 140 of 184 countries. According to the 2012 report of the GLOBOCAN Project, breast cancer accounts for 25% (1.67 million) of all new cancer cases [24]: our results demonstrate that the perception of its risk is the highest, and remains steady throughout the entire lifespan. Breast cancer affects women of all ages, with most cases diagnosed in women older than 50 years and women younger than 45 years only accounting for <10% of all cases. Our results confirm that breast cancer risk perception and screening behaviour are between the most important factors that must be addressed during gynaecological consultations [25]. This is true although how its risk varies during use of hormonal treatments is not fully understood [26,27], also in women with a family history [28].

Conversely, cervical cancer is most frequently diagnosed in women between the ages of 35 and 44, and for this reason it is not surprising that the correlation is more reported by premenopausal women in our study [19]. Cervical cancer is caused by HPV infection, and exposure to genital HPV is not independent of HC use [19]. Women using HC are more likely to be exposed to HPV than those using barrier contraceptive methods or not having sexual intercourse.

Numerous studies of interventions providing tailored information about cancer risk or possible screening, delivered by print or computer, have been conducted in the last decade. However, there is no evidence that the tailoring of these messages affects secondary prevention measures, such as the uptake of breast or uterine cervix cancer screening [29]. In conclusion, as consultation time is scarce, our messages surrounding hormone therapies must be focussed on the perceptions of the patients.

### Limitations

There were several limitations of this survey, including its cross-sectional study design and the simplified form of the questionnaire used in both pre- and post-menopausal women. In addition, our sample size calculation was able to recognize a true difference in means of 0.35 unit in some Likert Scale values between different groups, pre vs. postmenopausal women. However, it would be not large enough to enable the possible detection of negligible differences <0.35 point between different groups. Our results have not been corrected for family history of the presented diseases, for educational levels of women and for residual ovarian activity (in premenopausal women with premature ovarian failure) which could strongly affect their attitudes. The strengths of this study include the in-depth questions and the availability of a large volume of data from participants. Moreover, it is the first published study that evaluates similar questions and perceptions throughout a woman's whole life.

## Conclusions

The results of the present study should be used to guide our counselling activity when prescribing hormonal treatments in women. Addressing false myths and trying to overcome unjustified worries is one of the most important tasks in therapeutic prescription today. The benefit/risk calculus of HC and HT tips decidedly towards benefits for most women. Our data suggest that our patients are primarily interested in the effect of hormonal treatments on venous thrombosis, mood, breast and cervical cancer risk, and, later in life, impact on weight gain, breast cancer and ovarian cancer risk.

## Supplementary Material

Supplemental Material

## Data Availability

The data that support the findings of this study are available on request from the corresponding author, GG (to share upon reasonable request).
