# Supplementary material for: Attitudes of women towards products containing hormones (hormonal contraceptives or hormone therapy): what changes from pre to postmenopause?
Source: Ann Med. 2021 Jun 14;53(1):908–15. doi: 10.1080/07853890.2021.1938662 (PMC8205098; doi:10.1080/07853890.2021.1938662)
Supplement: Supplemental Material [file IANN_A_1938662_SM7105.pdf]

Pre-menopause

1. Are you taking/have you taken a hormonal contraceptive (pill, vaginal ring, patch)?

   ☐ Yes now ☐ Yes in the past ☐ No

2. If you answered yes:

- How long have you been taking it or how long have you used it before?
  …………………………………… months/years
- What mode of administration are you using or have you used? (oral, vaginal, patch)
  …………………………………………………………………………………………………
- Do you remember the name of the product?
  ……………………………………………………………………….

3. How much do you think that hormonal contraception (pill, vaginal ring or patch) can increase or reduce the risk of onset of the following cancers? (Considering a scale ranging from -5 to +5 where -5 = risk reduction, 0 = no influence, +5 = increased risk)

Breast cancer	-5	-4	-3	-2	-1	0	+1	+2	+3	+4	+5
Ovarian cancer	-5	-4	-3	-2	-1	0	+1	+2	+3	+4	+5
Colon cancer	-5	-4	-3	-2	-1	0	+1	+2	+3	+4	+5
Uterine body cancer	-5	-4	-3	-2	-1	0	+1	+2	+3	+4	+5
Uterine cervix cancer	-5	-4	-3	-2	-1	0	+1	+2	+3	+4	+5

4. How much do you think that hormonal contraception (pill, vaginal ring or patch) can influence the onset of the following diseases? (Considering a scale from -5 to +5, where -5 = reduces the onset, 0 = no influence, +5 = increases the onset)

Trombosis of the veins	-5	-4	-3	-2	-1	0	+1	+2	+3	+4	+5
Breast's cyst	-5	-4	-3	-2	-1	0	+1	+2	+3	+4	+5
Cardiovascular diseases	-5	-4	-3	-2	-1	0	+1	+2	+3	+4	+5
Depression	-5	-4	-3	-2	-1	0	+1	+2	+3	+4	+5

5. How much do you think hormonal contraception (pill, vaginal ring or patch) can improve or worsen the following symptoms? (Considering a scale from -5 to +5, where -5 = much worse, 0 = neither worse nor better, +5 = much better)

Headache	-5	-4	-3	-2	-1	0	+1	+2	+3	+4	+5
Weight gain	-5	-4	-3	-2	-1	0	+1	+2	+3	+4	+5
Decline of sexual desire	-5	-4	-3	-2	-1	0	+1	+2	+3	+4	+5
Vaginal dryness	-5	-4	-3	-2	-1	0	+1	+2	+3	+4	+5
Appetite	-5	-4	-3	-2	-1	0	+1	+2	+3	+4	+5
Mood swings	-5	-4	-3	-2	-1	0	+1	+2	+3	+4	+5

Post-menopause

1. Are you taking/have you taken hormone replacement therapy for menopause?

   ☐ Yes now ☐ Yes in the past ☐ No

2. If you answered yes:

- How long have you been taking it or how long have you used it before? ……………………………………… months/years
- What mode of administration are you using or have you used? (oral, transdermal, vaginal cream) ………
- Do you remember the name of the medicine? ……………………………………………………………………….

3. How much do you think that hormone replacement therapy for menopause can increase or reduce the risk of onset of the following cancers? (Considering a scale ranging from -5 to +5 where -5 = risk reduction, 0 = no influence, +5 = increased risk)

Breast cancer	-5	-4	-3	-2	-1	0	+1	+2	+3	+4	+5
Ovarian cancer	-5	-4	-3	-2	-1	0	+1	+2	+3	+4	+5
Colon cancer	-5	-4	-3	-2	-1	0	+1	+2	+3	+4	+5
Uterine body cancer	-5	-4	-3	-2	-1	0	+1	+2	+3	+4	+5
Uterine cervix cancer	-5	-4	-3	-2	-1	0	+1	+2	+3	+4	+5

4. How much do you think that hormone replacement therapy can influence the onset of the following diseases? (Considering a scale from -5 to +5, where -5 = reduces the onset, 0 = no influence, +5 = increases the onset)

Trombosis of the veins	-5	-4	-3	-2	-1	0	+1	+2	+3	+4	+5
Breast's cyst	-5	-4	-3	-2	-1	0	+1	+2	+3	+4	+5
Cardiovascular diseases	-5	-4	-3	-2	-1	0	+1	+2	+3	+4	+5
Depression	-5	-4	-3	-2	-1	0	+1	+2	+3	+4	+5

5. How much do you think hormone replacement therapy can improve or worsen the following symptoms? (Considering a scale from -5 to +5, where -5 = much worse, 0 = neither worse nor better, +5 = much better)

Headache	-5	-4	-3	-2	-1	0	+1	+2	+3	+4	+5
Weight gain	-5	-4	-3	-2	-1	0	+1	+2	+3	+4	+5
Decline of sexual desire	-5	-4	-3	-2	-1	0	+1	+2	+3	+4	+5
Vaginal dryness	-5	-4	-3	-2	-1	0	+1	+2	+3	+4	+5
Appetite	-5	-4	-3	-2	-1	0	+1	+2	+3	+4	+5
Mood swings	-5	-4	-3	-2	-1	0	+1	+2	+3	+4	+5

6. Have you ever used a hormonal contraceptive (pill, vaginal ring or patch) in the past?

   ☐ Yes☐ No

- What kind of hormonal contraceptive did you use? ………………………………………………………..
- How long did you take it? ……………………………………… months/years
- Do you remember the name of the contraceptive used? ……………………………………………………………………………….
